# Supplementary material for: Vibrio harveyi plasmids as drivers of virulence in barramundi (Lates calcarifer)
Source: PLoS One. 2025 May 19;20(5):e0319450. doi: 10.1371/journal.pone.0319450 (PMC12088062; doi:10.1371/journal.pone.0319450)
Supplement: S1 Appendix — (DOCX) [file pone.0319450.s001.docx]

*The following supplement accompanies the article*

***Vibrio harveyi* plasmids as drivers of virulence in barramundi (*Lates calcarifer*)**

Roisin Sullivan^1,2^, Joy A Becker^2^, Ruth N Zadoks^1^, Carola Venturini^1,3^, Ana I. S. Esteves^4#a^, Suresh Benedict^5^, Dani L Fornarino^5^ , Hannah Andrews^5^, God’spower R Okoh^5^, Vidya Bhardwaj^5#b^, Mark Sistrom^5^, Mark E Westman^1,4^, Nguyen Ngoc Phuoc^6^, Francisca Samsing^1*^

^1^ Sydney School of Veterinary Science, Faculty of Science, The University of Sydney, NSW, Australia

^2^ School of Life and Environmental Sciences, Faculty of Science, The University of Sydney, Camden, NSW, Australia

^3^ Westmead Institute for Medical Research, Westmead, NSW, Australia

^4^Elizabeth Macarthur Agricultural Institute, New South Wales Department of Primary Industries and Environment, Menangle, New South Wales, Australia

^5^ Berrimah Veterinary Laboratory, Department of Agriculture and Fisheries, Northern Territory Government, Berrimah, Northern Territory, Australia

^6^ Faculty of Fisheries, University of Agriculture and Forestry, Hue University, Hue City, Viet Nam

^#a^ Current address: School of Biomedical Engineering, Faculty of Engineering, The University of Sydney, Camperdown, NSW, Australia

^#b^ CSIRO Australian Centre for Disease Preparedness, Geelong, Victoria, Australia

* Corresponding author: Dr Francisca Samsing

email: francisca.samsingpedrals@sydney.edu.au

**S1 Appendix.** Supplemental methods accompanying **Section 2.3**

For the investigation of phenotypic characteristics of virulence in the *V. harveyi* isolates collected from moribund fish in Vietnam and the *V. harveyi* isolate TCFB-0558, several phenotypic assays were performed alongside infrared biotyping. The details of which are explained in full below with reference to source protocols where applicable. All isolates were grown from cryo-preserved aliquots on marine agar overnight at 28°C and passaged once more at 28°C prior to testing. For infrared biotyping, isolates were grown on marine agar form frozen aliquots and incubated for 24 hours at 28°C. A sub-culture was taken from this and incubated for 24 hours at 28°C prior to use for biotyping.

*Lipase and phospholipase*

Lipase and phospholipase activities were determined according to the protocol of Natrah (1). In brief, for lipase, marine agar was supplemented with 1% Tween-80 whilst for phospholipase, marine agar was supplemented with 1% sterile egg yolk emulsified in sterile saline. For each isolate, using a sterile disposable inoculating needle, a single colony was stabbed into the respective agar and incubated for up to 72 hours at either 22°C, 28°C or 34°C. Plates were observed every 24 hours for the development of opalescent zones around the colonies and the diameter of the zones were measured using a ruler at 48 hrs. Measurements from the other timepoints were excluded as only small < 0.5 mm zones were observed at 24 hours and by 72 hours, the majority of isolates had overgrown the plates at 28°C.

*Caseinase*

Caseinase activity was tested according to Yang (2). Media was prepared by mixing sterile double-strength marine agar (3% agar) with a sterile 4% skim-milk powder suspension in a 50:50 ratio. A single colony was stabbed into plates and incubated at 22°C, 28°C or 34°C for up to 72 hours. Plates were observed every 24 hours for the appearance of clearing zones around colonies and the diameter of the clearing zones was measured for each isolate at 48 hrs using a ruler. Measurements from the other timepoints were excluded as clearing zones were < 0.5 mm zones at 24 hours and by 72 hours, the isolates had overgrown the 28°C plates.

*Haemolysis*

Haemolytic activity was investigated using Columbia Sheep Blood agar plates (SBA) (ThermoFisher PP2133). Isolates were stabbed into SBA using disposable sterile inoculating needles and incubated at 22°C, 28°C or 34°C for up to 72 hours. Plates were checked every 24 hours and haemolytic clearing zones were classified as β-haemolytic (complete), α-haemolytic (partial) or non-haemolytic and their diameter measured using a ruler at 72 hours

*Urease*

Urease activity was assessed in broth culture using a modified protocol of Carson (3). Urease-specific media was prepared with 0.25 g of peptone, 0.25 g glucose, 3.75 g sodium chloride, 0.3 g disodium phosphate, 0.2 g potassium dihydrogen phosphate, 10 mL phenol red and 237.5 mL MilliQ water which was then autoclaved at 115°C for twenty minutes. A filtered urea stock (40% w/v in MilliQ water) was prepared at room temperature and 12.5 mL added to the liquid media and mixed before dispensing into sterile tubes. Once prepared, 3 colonies were collected from each isolate grown on marine agar at 28°C and inoculated into the urease broth. Broth tubes were then incubated at 22°C, 28°C or 34°C and a change in colour to pink/red at 48 hours was recorded as a positive result. Three replicates were performed for each isolate at each temperature.

*Swarming motility*

Swarming motility was assessed according to Rui (4) with minor modifications. A standardised suspension was prepared for use by inoculating 1 colony from a sub-culture of each isolate into marine broth (Difco 2216) and incubating overnight at 28°C. This suspension was then diluted in sterile PBS until an OD_600_ of 0.5 was reached prior to use for testing. Soft marine agar (0.3% agar) was prepared and 5 $\mu$L of the standardised suspensions for each isolate was dropped onto the centre of each plate (n =5 per isolate). Plates were allowed to air-dry inside the biosafety cabinet before being incubated at 22°C, 28°C or 34°C for 24 hours and the diameter of colony forming units was measured.

*Gelatinase*

Gelatinase activity was measured according to Yang, Anh (2). Media was prepared by adding 0.5% gelatine to marine agar prior to autoclaving. Plates were inoculated with 10 $\mu$L of the standardised suspension of each isolate and incubated at 22°C, 28°C or 34°C. Plates were incubated for 72 hours at their respective temperatures, at which point plates were flooded with saturated ammonium sulphate ((NH_4_)_2_SO_4_). After 20 minutes, the ammonium sulphate was decanted, and the diameter of clearing zones was measured using a ruler.

*Biofilm formation*

Biofilm activity was assessed using the Dojindo Biofilm Formation Assay Kit (B601, Dojindo Laboratories, Japan). The standardised suspensions of each isolate were added in triplicate to a sterile 96-well plate (180 mL/well) with the associated peg-lid and incubated for 48 hours at 22°C, 28°C or 34°C. After incubation, each plate was washed twice with 200 $\mu$L PBS followed by the addition of 200 $\mu$L Crystal Violet. After 30 minutes of incubation at room temperature, the plates were washed twice with 200 $\mu$L PBS and eluted in 200 $\mu$L of absolute ethanol. Following a second incubation at room-temperature for 20 minutes, absorbance was measured at 590 nm on a SpectraMax iD3 plate reader (Molecular Devices, California, USA). To account for background level absorbance, the average absorbance of the negative marine broth control was subtracted from the average absorbance measurements of each isolate at each temperature.

*Fourier-Transform Infrared Spectroscopy*

An alternative to whole-genome sequencing is Fourier-transform infrared spectroscopy (FT-IR) which allows for cost-effective and rapid fingerprinting of the isolates based on bacterial cell composition [5]. For FT-IR typing using the Infrared (IR) Biotyper (IRBT) system (Bruker Daltonics, Germany), isolates were resuscitated from cryo-preservation and incubated for 24 hours at 28°C on marine agar. From this culture, a new passage was made on marine agar and incubated for 24 hours at 28°C. This sub-culture was then used for infrared biotyping. Briefly, one overloaded 1 $\mu$L loop of culture was resuspended in 50$\mu$L of 70% ethanol and homogenized in 1.5 mL tubes containing metal rods (Bruke Daltonics, Germany). Following this, 50 $\mu$L of sterile nuclease-free water was added to the tubes and vortexed. Once homogeneous, 15 $\mu$L of the suspension was spotted onto a 96-well IRBT silicon plate (Bruker Daltonics, Germany) in triplicate and allowed to dry at room-temperature for 1 hour. Each plate also included two IR test standards (IRTS1 and IRTS2), spotted in duplicate as 10 $\mu$L droplets for quality control. Three biological replicates were performed (on separate days) with three technical replicates per biological replicate for each isolate. Measurements were performed under the default settings of the IRBT system and analysed using the IRBT software.

**Supplemental References:**

1. Natrah FMI, Ruwandeepika HAD, Pawar S, Karunasagar I, Sorgeloos P, Bossier P, et al. Regulation of virulence factors by quorum sensing in *Vibrio harveyi*. Vet Microbiol. 2011;154(1):124-9. doi: 10.1016/j.vetmic.2011.06.024.

2. Yang Q, Anh ND, Bossier P, Defoirdt T. Norepinephrine and dopamine increase motility, biofilm formation, and virulence of *Vibrio harveyi*. Front Microbiol. 2014;5:584. doi: 10.3389/fmicb.2014.00584.

3. Carson J, Higgins M, Wilson T, Gudkovs N, Bryant T. Identification of *Vibrionaceae* from Australian aquatic animals using phenotypic and PCR procedures. Australia and New Zealand Standard Diagnostic Procedure. 2009.

4. Rui H, Liu Q, Ma Y, Wang Q, Zhang Y. Roles of LuxR in regulating extracellular alkaline serine protease A, extracellular polysaccharide and mobility of *Vibrio alginolyticus*. FEMS Microbiol Lett. 2008;285(2):155-62. doi: 10.1111/j.1574-6968.2008.01185.x.

5. Baker MJ, Trevisan J, Bassan P, Bhargava R, Butler HJ, Dorling KM, et al. Using Fourier transform IR spectroscopy to analyze biological materials. Nat Protoc. 2014;9(8):1771-91. doi: 10.1038/nprot.2014.110.
