# Supplementary figures and images for: Vibrio harveyi plasmids as drivers of virulence in barramundi (Lates calcarifer)
Source: PLoS One. 2025 May 19;20(5):e0319450. doi: 10.1371/journal.pone.0319450 (PMC12088062; doi:10.1371/journal.pone.0319450)

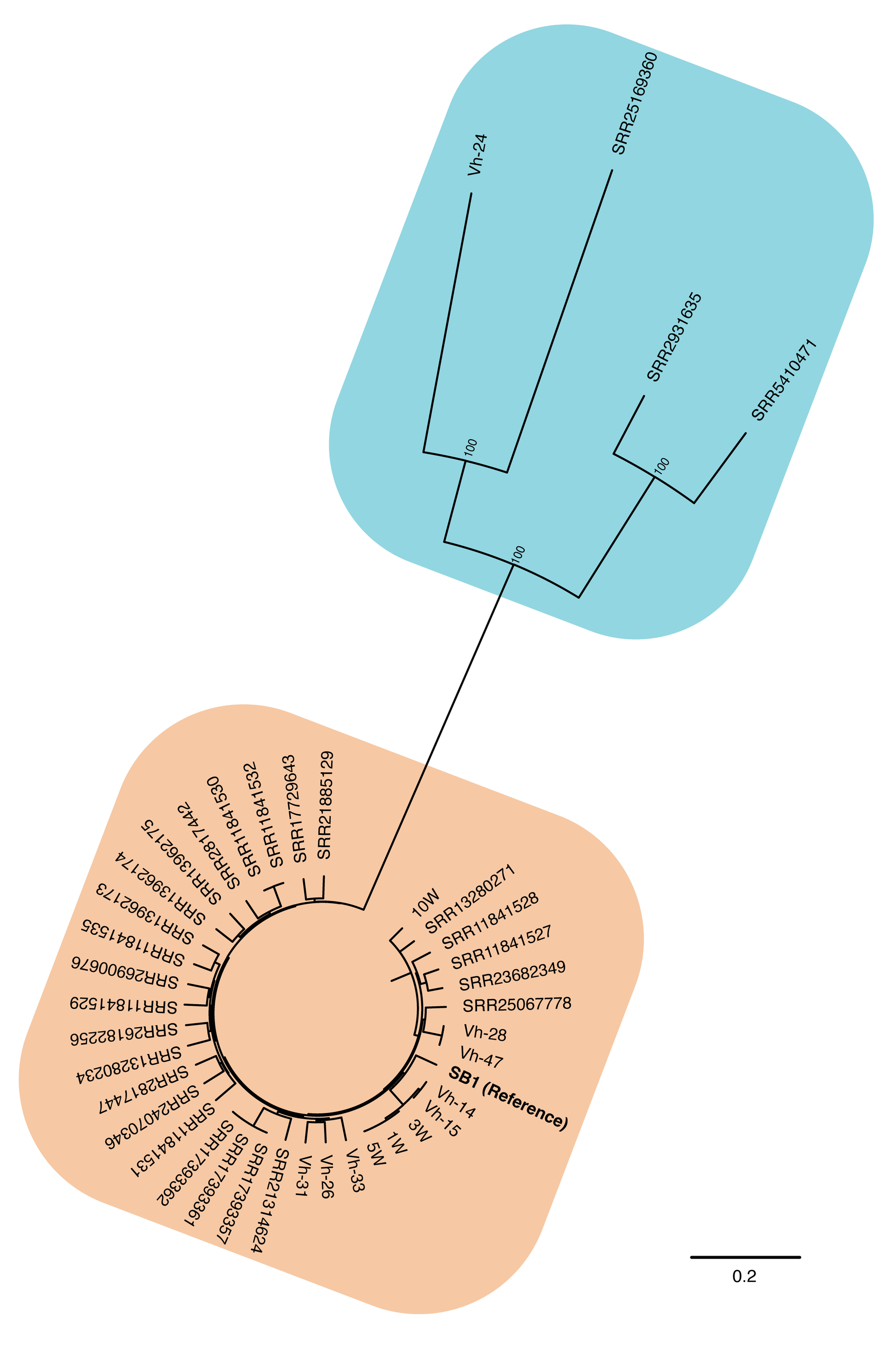

Supplement: S1 Fig — SNP-based phylogeny for the 12 isolates sequenced with short-read sequencing in this study (labelled Vh- or -W), the twenty-eight sequences downloaded from the SRA labelled with their unique SRR number and the Vibrio harveyi reference genome (SB1, NCBI: PRJNA972608). A distinct outgroup of four isolates (shaded in blue) formed from the other isolates. Vh-24 had an 97.3% ANI to the Vibrio campbellii reference genome (BoB-53, NCBI: PRJNA429202). (TIF) [file pone.0319450.s003.tif]
